# Supplementary material for: Proteolysis of histidine kinase VgrS inhibits its autophosphorylation and promotes osmostress resistance in Xanthomonas campestris
Source: Nat Commun. 2018 Nov 15;9:4791. doi: 10.1038/s41467-018-07228-4 (PMC6237974; doi:10.1038/s41467-018-07228-4)
Supplement: Supplementary file 1 — Supplementary Information [file 41467_2018_7228_MOESM1_ESM.pdf]

Supplementary Information

Proteolysis of histidine kinase VgrS inhibits its autophosphorylation and promotes osmostress resistance in *Xanthomonas campestris*

Deng et al.

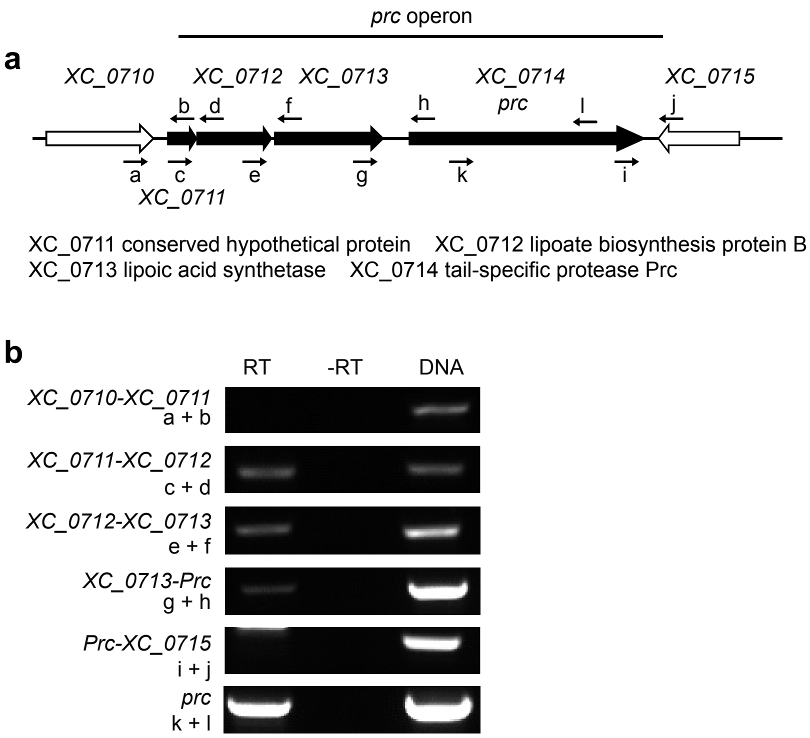

Supplementary Fig. 1 *prc* is located in an operon containing four genes. **a** Organization of the *prc* operon. Upper panel: location of genes in the genome of *X. campestris* pv. *campestris* 8004. Large arrows indicate genes and their transcriptional directions. Alphabets a to j indicate the location of primers used to amplify the potential intergenic transcripts. Lower panel: protein products of the genes. **b** RT-PCR assay of intergenic regions between *XC_0710* to *XC_0715*. Primers were used to amplify the cDNA of intergenic transcripts. Amplification of *prc* cDNA was used as positive control. RT: RT-PCR. –RT: negative control, using cDNA which was synthesized without reverse transcriptase. DNA: bacterial genome DNA using as the positive control. The experiment was repeated three times.

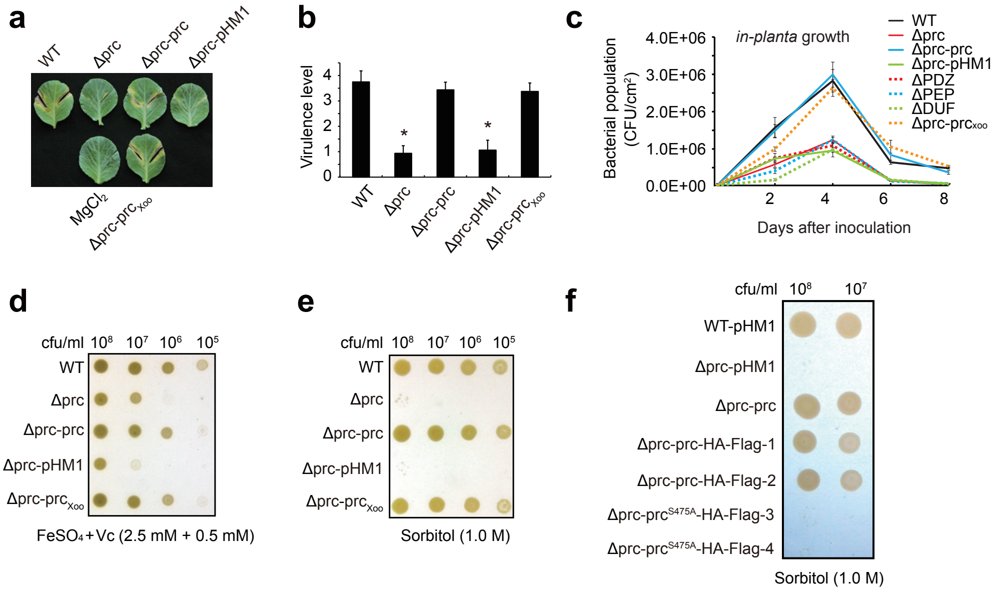

Supplementary Fig. 2 Prc function is evolutionarily conserved between *X. campestris* pv. *campestris* and *X. oryzae* pv. *oryzae*. **a** and **b** Bacterial virulence against host plant *B. oleraceae* cv. Jingfeng No. 1. Virulence scores were estimated 10 d after inoculation. Sterile $MgCl_2$ (10 mM) was inoculated as a negative control. **a** Lesions in plant leaves. **b** Virulence scores of bacterial strains as shown in **a**. The virulence levels were estimated by a semi-quantitative standard. Genetic complementary by *in trans* providing a *prc* of *X. oryzae* pv. *oryzae* (prc_{Xoo}) fully restored the phenotypic deficiencies of *prc* deletion mutant of *X. campestris* pv. *campestris*. Asterisk indicates significant difference relative to the WT strain ($P < 0.05$, $n = 12$). **c** *in-planta* growth assay of bacterial strains. Bacterial cells were infiltrated into plant leaves and populations were measured. For each data point, $n = 6$. **d** Bacterial resistance to iron stress. Bacterial strains were grown on NYG agar containing 2.5 mM $FeSO_4$ plus 0.5 mM vitamin C for 72 h under 28°C. **e** Bacterial resistance to osmostress. Bacterial strains were grown on NYG agar containing 1.0 M sorbitol for 72 h under 28°C. cfu: colony-forming unit. In **c** and **d**, each experiment was repeated three times. **f** C-terminal tags did not affect bacterial resistance to sorbitol stress. The growth of recombinant strains of Δprc-prc-HA-Flag or Δprc-prc^{S475A}-HA-Flag was compared with WT, Δprc, and Δprc-prc. Strains were grown on NYG agar containing 1.0 M sorbitol for 72 h under 28°C. **d**-**f**, each experiment was repeated for three times.

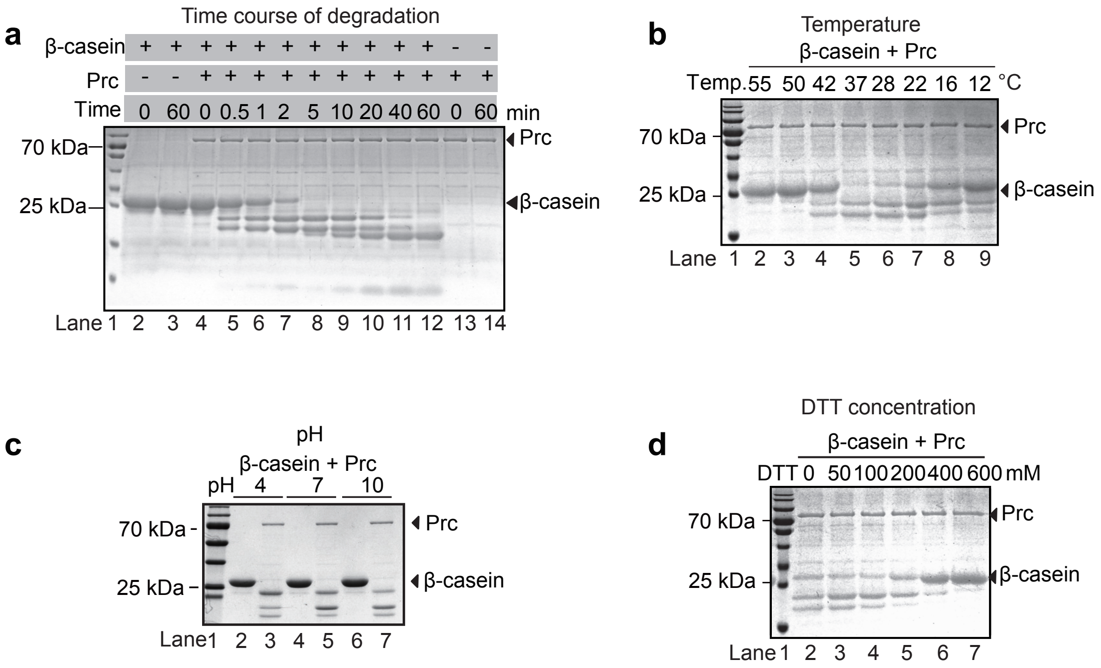

Supplementary Fig. 3 Impact of environmental factors on Prc activity. **a** Time course of Prc activity in degrading β-casein. **b** Impact of different temperature on the Prc activity. **c** Impact of different pH value on the Prc activity. **d** Impact of different DTT concentrations on the Prc activity. In these experiments, β-casein (40 μM) and Prc (1 μM) proteins were co-incubated. The reactions were stopped and analyzed by SDS-PAGE with Coomassie brilliant blue staining. Each experiment was repeated three times.

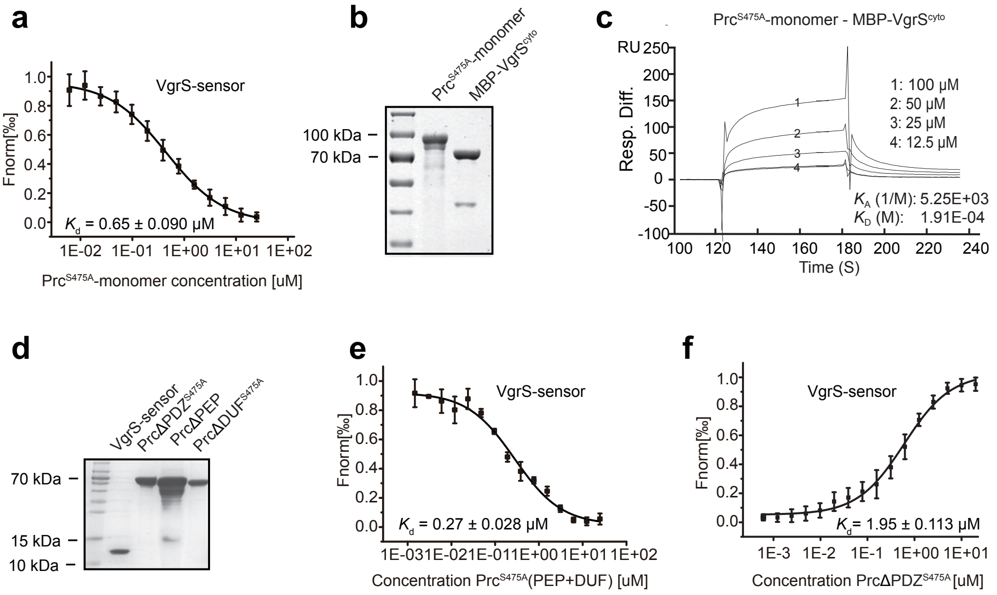

Supplementary Fig. 4 Identification of Prc region which binds VgrS sensor. **a** Microscale thermophoresis (MST) analysis revealed that VgrS sensor binds Prc monomer. VgrS sensor was labelled and the titrations of Prc^{S475A} monomer ranged from 0.006 to 25 μM. The solid curve is the fit of the data points to the standard KD-Fit function. Black bars represent standard deviations. K_d, dissociation constant. The experiment was repeated three times. **b** Purification of Prc monomer and cytosolic region of VgrS (MBP-$VgrS^{cyto}$). The proteins were separated by SDS-PAGE before staining with Coomassie brilliant blue. **c** Cytosolic region of VgrS did not bind Prc monomer. Surface plasmon resonance (SPR) was used to estimate kinetics of protein interactions. MBP-$VgrS^{cyto}$ protein was trapped on a sensor CM5 chip and various concentrations of Prc were injected at a flow-rate of 30 μl/min at 25°C. Data were analyzed using a model for a single set of identical binding sites. Binding kinetics of Prc-VgrS sensor interaction. K_A: equilibrium association rate constant. K_D: equilibrium dissociation rate constant. **d** Purification of truncated Prc^{S475A} proteins. Prc^{S475A} protein without peptidase, PDZ and DUF domains, respectively, was purified. The proteins were separated by SDS-PAGE before staining with Coomassie brilliant blue. **e** and **f** MST analyses revealed that VgrS sensor binds Prc^{S475A} proteins with peptidase and DUF domains. **e** Binding affinity between VgrS sensor and Prc^{S475A} (PEP + DUF). **f** Binding affinity between VgrS sensor and PrcΔPDZ^{S475A}. The condition of MST assays is similar to that described in **a**. Each experiment was repeated three times.

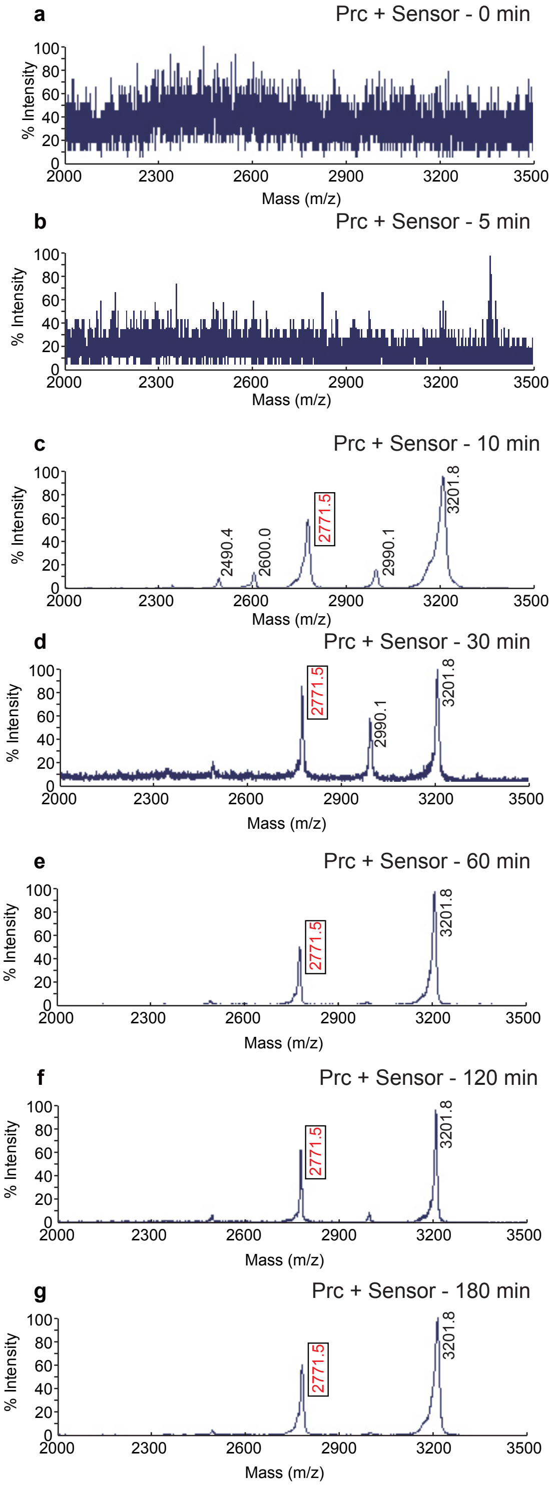

Supplementary Fig. 5 The MALDI-TOF-MS spectra analysis. **a-g** VgrS sensor was digested by Prc with indicated time and detected on AB Sciex 5800 MALDI-TOF TOF mass spectrometer in the positive ion mode over the *m/z* range from 2000 to 3500. Spectra showed the relative intensities with a zoom on the mass range.

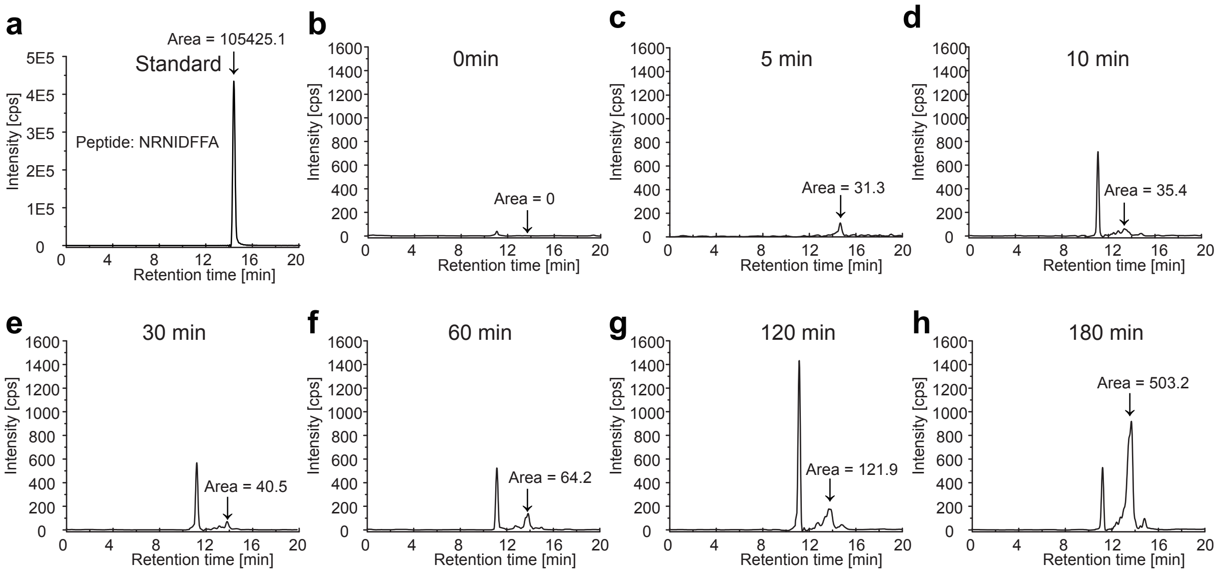

Supplementary Fig. 6 Accumulation of the cleaved, N-terminal peptide (2nd-9th aa.) of VgrS triggered by Prc proteolysis. **a** QTRAP LC–MS/MS analysis of chemically synthesized peptide (2nd-9th aa., NRNIDF-FA) standard. **b-h** Identification and quantification of relative content of the proteolytic peptides at different reaction time. Full-length VgrS embedded in the inverted membrane vesicles was mixed with Prc and co-incubated at the indicating time, equivalent volume of the mixer was collected and subjected to liquid nitrogen for fixing the proteolytic status. After filtration with 3K Ultratfiltration devices, the peptides below 3 kDa were concentrated and then used for QTRAP LC–MS/MS analysis. The relative levels of the peptide were determined with Analyte Peak Area (counts).

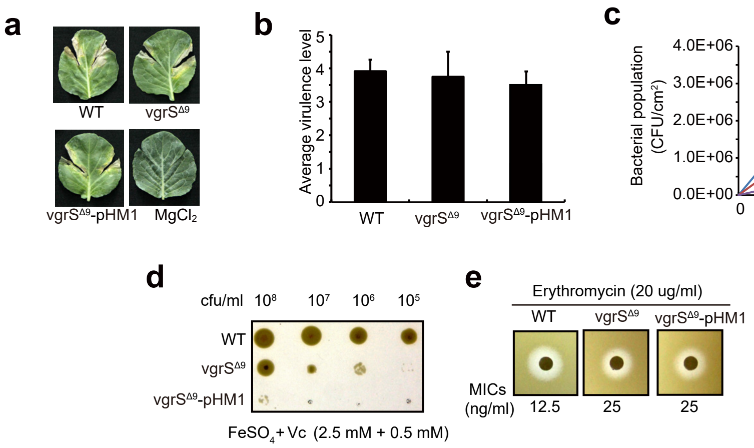

Supplementary Fig. 7 Phenotypic characterization of mutant strain vgrS$^{\Delta 9}$. vgrS$^{\Delta 9}$ was constructed by amino acids deletion in the N-terminus of *vgrS*. **a** The virulence assay of vgrS$^{\Delta 9}$. Bacterial strains were inoculated onto the leaves of *B. oleraceae* cv. Jingfeng No. 1. Virulence scores were estimated 10 d after inoculation. Sterile 10 mM $MgCl_2$ was inoculated as a negative control. **b** Virulence scores of bacterial strains. The virulence levels of bacterial strains were estimated using a semi-quantitative standard. Asterisks indicate significant differences compared with the WT strain (Student *t*-test, $P < 0.05$, $n = 12$). **c** *in-planta* growth assay of bacterial strains. For each data point, $n = 6$. **d** The sensitivity of iron stress of vgrS$^{\Delta 9}$. Bacterial strains were grown on NYG agar containing 2.5 mM $FeSO_4$ plus 0.5 mM vitamin C for 72 h at 28°C. **e** The sensitivity of erythromycin of vgrS$^{\Delta 9}$. The inhibitory zones of antibiotics are shown, and the minimal inhibitory concentrations (MICs) of antibiotics were measured and are listed below. In **d** and **e**, the experiments were repeated three times.

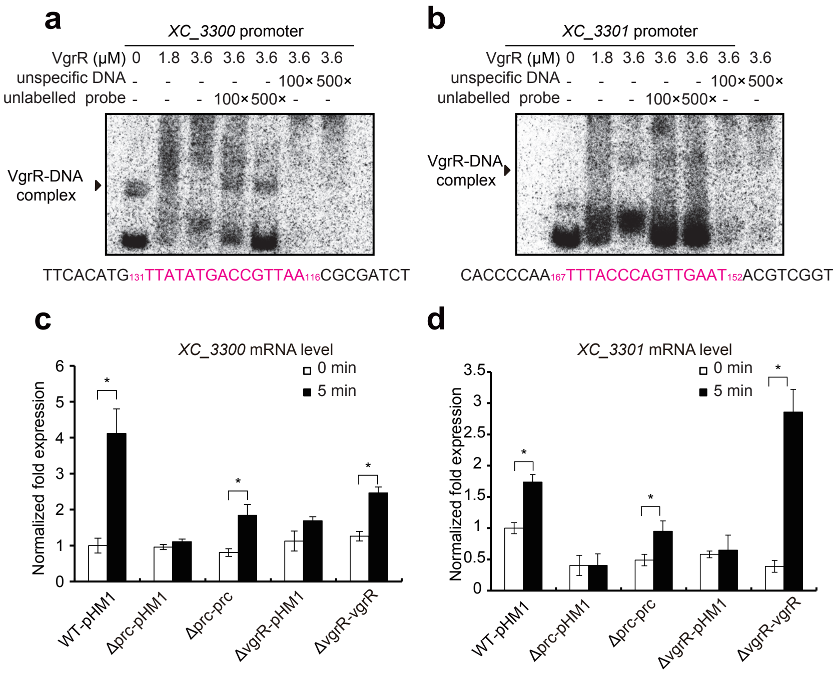

Supplementary Fig. 8 *prc* positively modulates the VgrR regulon. **a** and **b** EMSA revealed that VgrR directly bound the promoter region of *XC_3300* and *XC_3301.* PCR products of the promoter regions were labelled with [γ-^{32}P]ATP and used as DNA probes. Unlabelled DNA and non-specific DNA were used as competitors. The sequence of DNA probe was shown below with VgrR-binding motif in magenta. Numbers indicate the location relative to the translation initiation site. Each experiment was repeated two times. Triangles indicate VgrR-DNA complexes. **c** and **d** The *prc* mutation caused decrease in the transcription level of X*C_3300* and *XC_3301* when bacterial strains were grown under osmostress. qRT-PCR was used to quantify the mRNA amounts of these genes under different bacterial background before and after osmostress stimulation (1.0 M sorbitol, 5 min). The expression of tmRNA was used as an internal control. A representative of three independent experiments is shown. In **c** and **d**, Error bars indicate the standard deviations. Asterisks indicate significant differences of strains before and after osmostress (Student's *t*-test, $P < 0.05$).

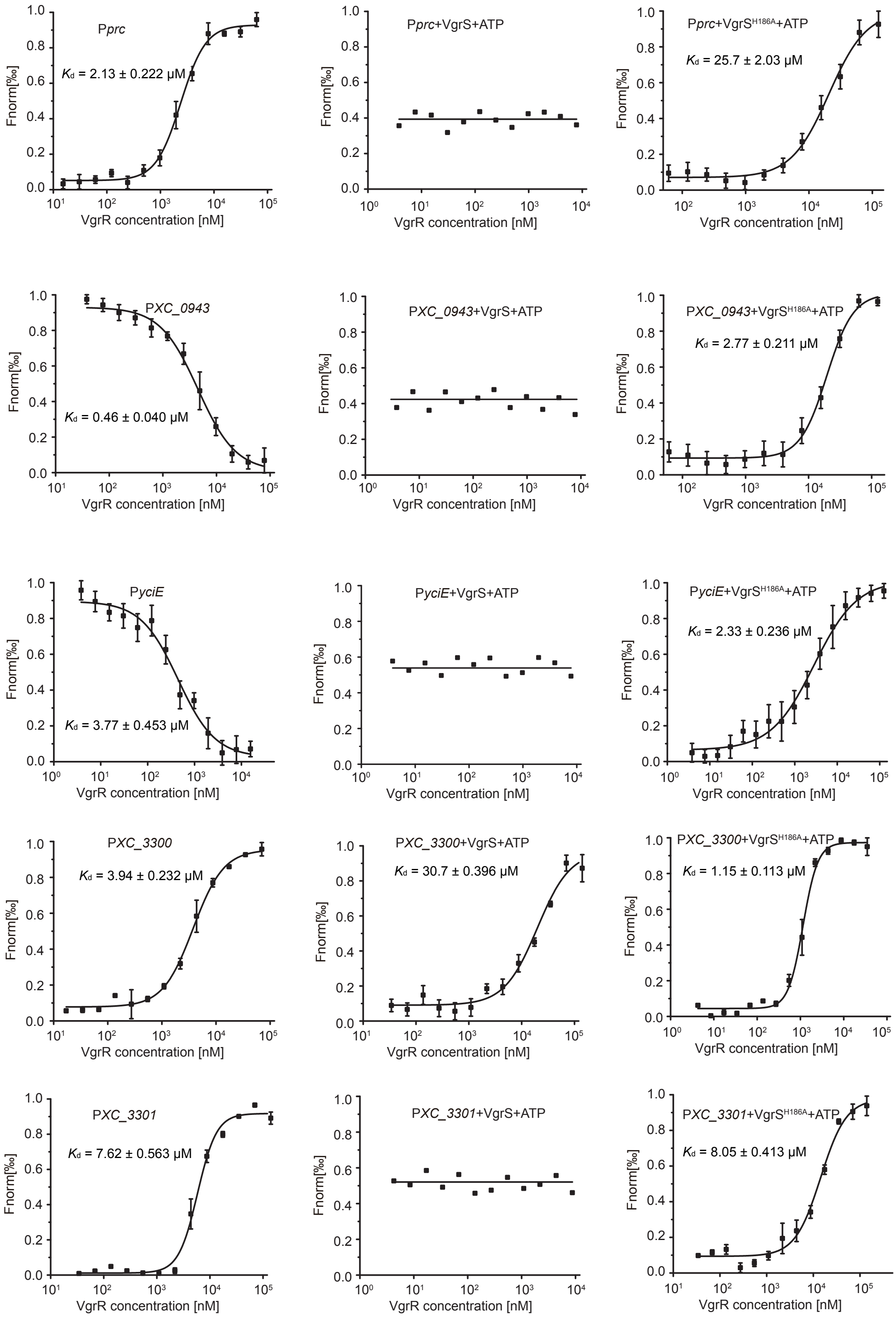

Supplementary Fig. 9 Phosphorylation of VgrR decreases the VgrR–DNA binding affinity. **a**–**e** Double-stranded DNA probes were labelled by 5'-FAM and microscale thermophoresis was used to measure the VgrR–DNA binding affinity. Binding affinities between the VgrR and promoter regions of *prc*, *XC_0943*, *XC_2164*, *XC_3300* and *XC_3301*, respectively. Left panels: Binding affinity of the unphosphorylated VgrR and DNA probes. Middle panels: Binding affinity of the phosphorylated VgrR and DNA probes. VgrR was phosphorylated by VgrS in the presence of ATP, and could not be phosphorylated by VgrS^{H186A}. Right panels: Negative controls. Each experiment was repeated three times. K_d, dissociation constant.

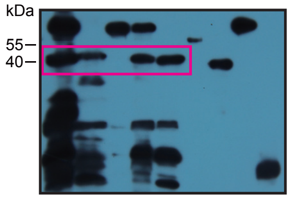

Fig 1b α-VgrS

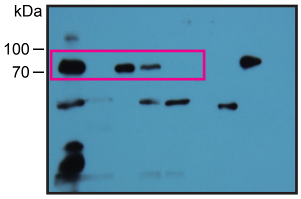

Fig 1b α-Prc

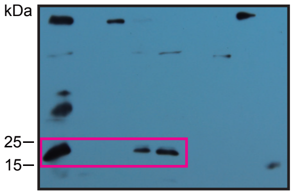

Fig 1b α-HppK

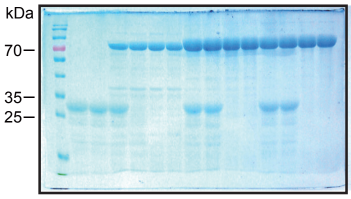

Fig 2

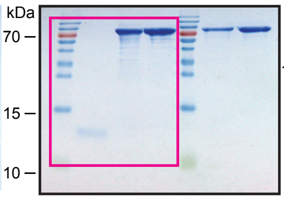

Fig 3c CBB

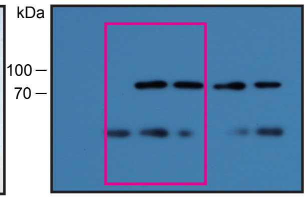

Fig 3c α-Prc

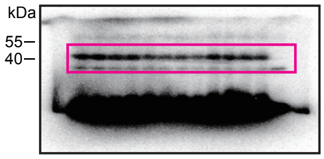

Fig 4a Autokinase assay

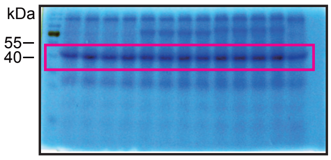

Fig 4a CBB

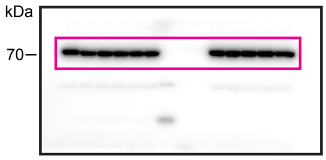

Fig 4b Autokinase assay

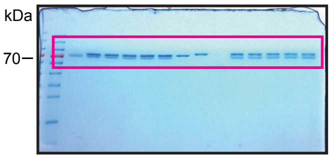

Fig 4b CBB

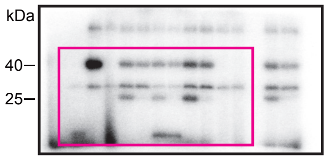

Fig 4c Autokinase assay

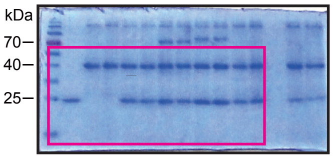

Fig 4c CBB

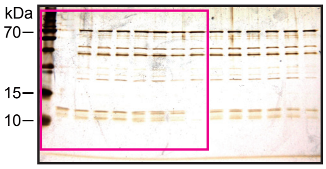

Fig 4d Silver staining

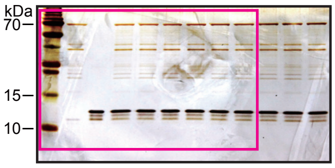

Fig 4e Silver staining

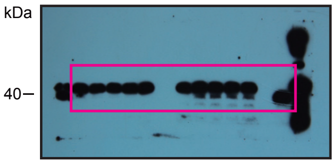

Fig 4f α-VgrS

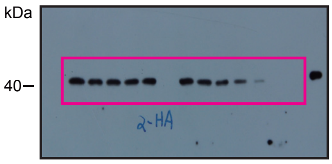

Fig 4f α-HA

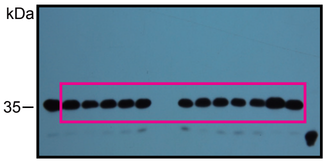

Fig 4f α-RNAP

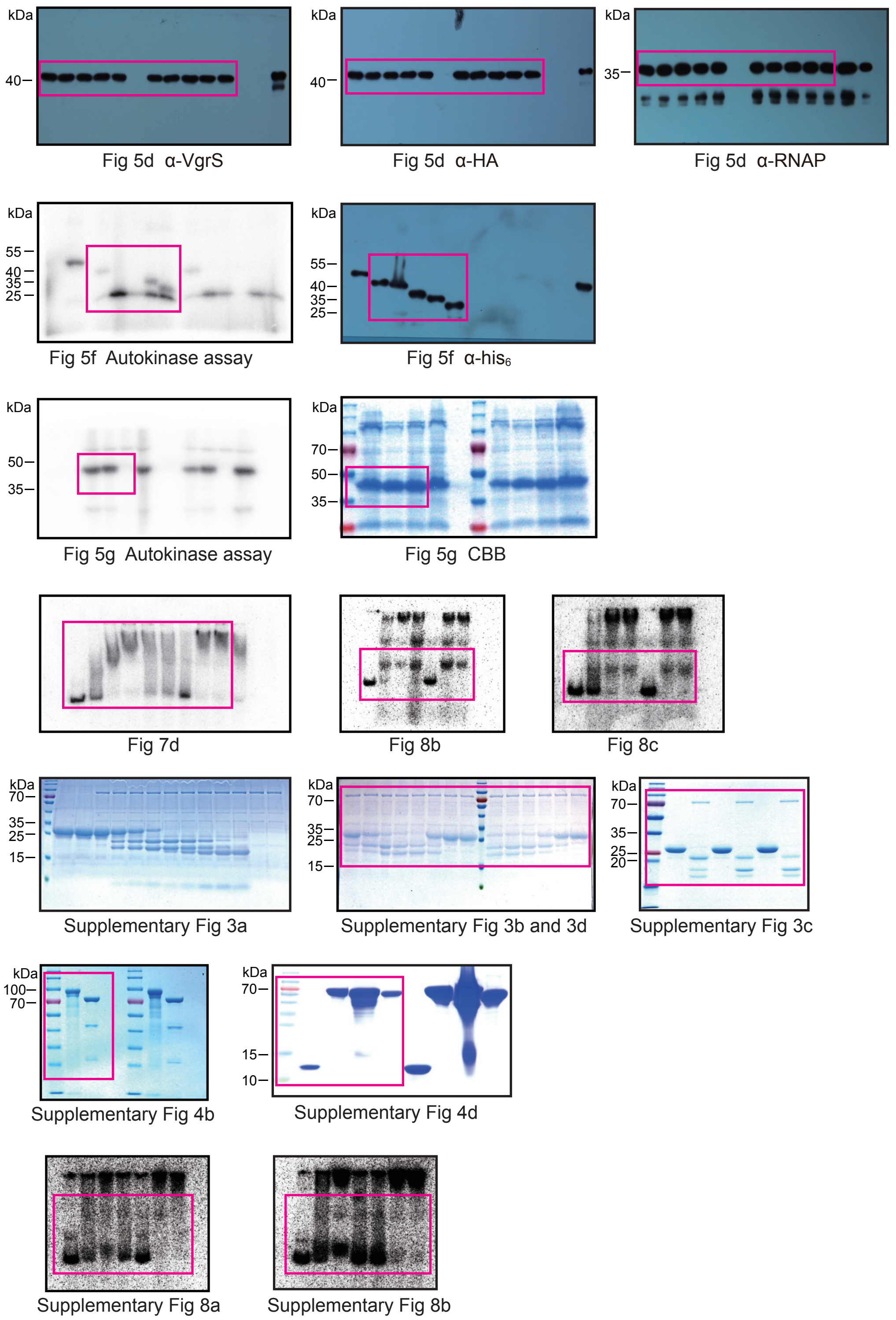

Supplementary Fig. 10 Full images of the Western blots and protein gels used in the study. The magenta frames indicate the cropped parts. CBB: Coomassie bright blue staining.These images include results of Western blotting, in vitro phosphorylation assay, EMSA and CBB staining of the proteins.

Supplementary Table 1. Bacterial strains and plasmids used in this study

Names	Genotype or relevant properties	Source
**Bacterial Strains**		
Escherichia coli strains		
E. coli DH5α	*fhuA2* Δ*(argF-lacZ)U169 phoA glnV44 Φ80* Δ*(lacZ)M15 gyrA96 recA1 relA1 endA1 thi-1 hsdR17*	Lab collection
E. coli BL21(DE3)	*E. coli B F⁻ dcm ompT hsd(r_B- m_B-) gal λ(DE3)*	Lab collection
Xanthomonas campestris pv. *campestris* strains		
WT	*Xcc* 8004 wild type strain (WT), Rif^r	Lab collection
WT-pHM1	WT containing a blank pHM1 vector, Rif^r, Sp^r	Lab collection
M0120	ΔvgrR, marker-exchange mutant of *XC_1049*(*vgrR*), replacement *vgrR* with a tetracycline resistance genes, Rif^r, Tc^r	Wang et al, 2016[1]
M0121	ΔvgrR-pHM1, ΔvgrR containing a pHM1 vector, Rif^r, Sp^r	Wang et al, 2016[1]
M0122	ΔvgrS, *XC_1050* (*vgrS*) in-frame deletion mutant, Rif^r	Wang et al, 2016[1]
M0123	ΔvgrS-pHM1, ΔvgrS containing a blank pHM1 vector, Rif^r, Sp^r	Wang et al, 2016[1]
M0124	ΔvgrR-vgrR, genetic complementary strain. Δ*vgrR* containing a pHM1::vgrR vector, Rif^r, Sp^r	Wang et al, 2016[1]
M0125	ΔvgrS-vgrS, genetic complementary strain. ΔvgrS containing a pHM1::*vgrS* vector, Rif^r, Sp^r	Wang et al, 2016[1]
M0134	WT-vgrR-his_6, WT containing a pHM1::vgrR vector with the C terminal of VgrR addition of hexa-histidine tag, Rif^r	Wang et al, 2016[1]
M0136	ΔvgrR-$vgrR^{D51A}$, genetic complementary strain. Δ*vgrR* containing a pHM1::$vgrR^{D51A}$ vector, Rif^r, Sp^r	Wang et al, 2016[1]
M0137	ΔvgrS-$vgrS^{H186A}$, genetic complementary strain. ΔvgrS containing a pHM1::$vgrS^{H186A}$ vector, Rif^r, Sp^r	Wang et al, 2016[1]
M0190	Δprc, *XC_0714*(*prc*) in-frame deletion mutant, Rif^r	This study
M0191	ΔPEP, in-frame deletion mutant of PEP domain, Rif^r	This study
M0192	ΔPDZ, in-frame deletion mutant of PDZ domain, Rif^r	This study
M0193	ΔDUF, in-frame deletion mutant of DUF domain, Rif^r	This study
M0194	Δprc-prc, genetic complementary strain. Δprc containing a pHM1::*prc* vector, Rif^r, Sp^r	This study
M0195	Δprc-pHM1, Δprc containing a blank pHM1 vector, Rif^r, Sp^r	This study

M0196	Δprc-prc$_{Xoo}$, heterogenetic complementary strain. Δprc containing a pHM::*prc*$_{Xoo}$ vector, Rif^r, Sp^r	This study
M0197	Δprc-prc-HA-FLAG, strain for tandem affinity Co-IP. Δprc containing a pHM1::*prc*-HA-Flag, Rif^r, Sp^r	This study
M0198	Δprc-prc^{S475A}-HA-FLAG, strain for tandem affinity Co-IP. Δprc containing a pHM1::*prc*-HA-Flag with a S475A mutation, Rif^r, Sp^r	This study
M0199	ΔvgrS-vgrS$^{A9G-Q10A}$, genetic complementary strain. ΔvgrS containing a pHM1::*vgrS*$^{A9G-Q10A}$ vector, Rif^r, Sp^r	This study
M0200	vgrS^{Δ9}*, XC_1050* (*vgrS*) with 2nd to 9th aa. being deleted, Rif^r	This study
M0201	vgrS^{Δ9}-pHM1, ΔvgrS^{Δ9} containing a blank pHM1 vector, Rif^r, Sp^r	This study
M0202	Δprc-vgrS^{Δ9}, *prc* and *vgrS*^{Δ9} double in-frame deletion mutant, Rif^r	This study
M0203	ΔprcΔvgrS, *prc* and *vgrS* double in-frame deletion mutant, Rif^r	This study
M0204	ΔprcΔvgrS-prc, ΔprcΔvgrS containing a pHM1::*prc* vector, for epistatic analysis, Rif^r, Sp^r	This study
M0205	ΔprcΔvgrS-vgrS, ΔprcΔvgrS containing a pHM1::*vgrS* vector, for epistatic analysis, Rif^r, Sp^r	This study
M0206	ΔprcΔvgrS-pHM1, ΔprcΔvgrS containing a pHM1 vector, for epistatic analysis, Rif^r, Sp^r	This study
M0207	ΔvgrS-3HA-vgrS, ΔvgrS containing a pHM1:: 3HA-*vgrS*^{4}. Coding sequence of the HA tag was inserted into the site between 3rd and 4th amino acid of VgrS, for detection the amount of VgrS protein *in vivo*, Rif^r, Sp^r	This study
M0208	ΔvgrS-vgrS$^{HA-A9G-Q10A}$, ΔvgrS containing a pHM1:: *vgrS*$^{HA\text{-}A9G\text{-}Q10A}$ with N terminal the third amino acid of VgrS insertion of a HA tag and with A9G, Q10A double mutation, for detection the amount of VgrS protein *in vivo*, Rif^r, Sp^r	This study
M0209	ΔprcΔvgrS-vgrS^{HA}, ΔprcΔvgrS containing a pHM1:: *vgrS*^{HA} with N terminal the third amino acid of VgrS insertion of a HA tag, for detection the amount of VgrS protein *in vivo*, Rif^r, Sp^r	This study
M0210	ΔprcΔvgrS-vgrS$^{HA-A9G-Q10A}$, ΔprcΔvgrS containing a pHM1:: *vgrS* $^{HA\text{-}A9G\text{-}Q10A}$ with N terminal the third amino acid of VgrS insertion of a HA tag and with A9G, Q10A double mutation, for detection the amount of VgrS protein *in vivo*, Rif^r, Sp^r	This study
M0211	ΔprcΔvgrR*, prc* and *vgrR* double in-frame deletion mutant, Rif^r	This study
M0212	ΔprcΔvgrR-prc, ΔprcΔvgrR containing a pHM1::prc vector, for epistatic analysis ,Rif^r, Sp^r	This study
M0213	ΔprcΔvgrR-vgrR, ΔprcΔvgrR containing a pHM1::vgrR vector, for epistatic analysis ,Rif^r, Sp^r	This study
M0214	ΔprcΔvgrR-pHM1, ΔprcΔvgrR containing a pHM1 vector, for epistatic analysis ,Rif^r, Sp^r	This study
M0215	ΔprcΔvgrR-vgrR^{D51A}, ΔprcΔvgrR containing a pHM1:: vgrR^{D51A} vector, for epistatic analysis ,Rif^r, Sp^r	This study

M0216	Δprc-vgrR-his_6, Δprc containing a pHM1::*vgrR* vector with the C terminal of VgrR addition of hexa-histidine tag, Rif^r	This study
M0217	ΔvgrS-3×Flag-vgrS, ΔvgrS containing a pHM1:: 3×Flag-*vgrS*. Coding sequence of the 3×Flag tag was inserted into the site between 1st and 2nd amino acid of VgrS, for detection the amount of VgrS protein *in vivo*, Rif^r, Sp^r	This study
M0218	ΔprcΔvgrS-$vgrS^{3×Flag}$, ΔprcΔvgrS containing a pHM1:: $vgrS^{3×Flag}$. Coding sequence of the 3×Flag tag was inserted into the site between 1st and 2nd amino acid of VgrS, for detection the amount of VgrS protein *in vivo*, Rif^r, Sp^r	This study
M0219	Δ0943, *XC_0943* in-frame deletion mutant, Rif^r	This study
M0220	Δ2164, *XC_2164* in-frame deletion mutant, Rif^r	This study
M0221	Δ0690, *XC_0690* in-frame deletion mutant, Rif^r	This study
M0222	Δ3300, *XC_3300* in-frame deletion mutant, Rif^r	This study
M0223	Δ3301, *XC_3301* in-frame deletion mutant, Rif^r	This study
M0224	Δ3576, *XC_3576* in-frame deletion mutant, Rif^r	This study
**Plasmids**		
pK18mobsacB	*sacB, lacZα*, Km^r/mobilizable *E. coli* suicide vector, allows selection for double-crossover	Lab collection
pHM1	Broad host range vector with pUC19 polylinker, used as the complementary vector, Sp^r	Lab collection
pET30a (+)	Protein expression vector, Kan^r	Novagen
pET30a-Prc	pET30a::Prc, expressing full length Prc_{Xcc}, Kan^r	This study
pET30a-Prc^{S475A}	pET30a::Prc^{S475A}, expressing full length Prc_{Xcc} with active site mutation S475A, Kan^r	This study
pET30a-Prc^{K500A}	pET30a::Prc^{K500A}, expressing full length Prc_{Xcc} with active site mutation K500A, Kan^r	This study
pET30a-PDZ	pET30a::PDZ, expressing PDZ domain of Prc_{Xcc}, Kan^r	This study
pET30a-PEP	pET30a::PEP, expressing PEP domain of Prc_{Xcc}, Kan^r	This study
pET30a-PEP^{S475A}	pET30a::PEP^{S475A}, expressing PEP domain of Prc_{Xcc} with active site mutation S475A, Kan^r	This study
pET30a-DUF	pET30a::DUF, expressing DUF domain of Prc_{Xcc}, Kan^r	This study
pET30a-PDZ+PEPΔ20	pET30a::PDZ+PEP, expressing PDZ and PEP domain of Prc_{Xcc}, Kan^r	This study
pET30a-PDZ+PEPΔ20^{S475A}	pET30a::PDZ+PEP^{S475A}, expressing PDZ and PEP domain of Prc_{Xcc} with active site mutation S475A,	This study

	Kan^r	
pET30a-PrcΔPDZ	pET30a::Prc, expressing full length Prc_{Xcc} with PDZ domain deletion, Kan^r	This study
pET30a-PrcΔPDZ^{S475A}	pET30a:: Prc^{S475A}, expressing full length Prc_{Xcc} with PDZ domain deletion and active site mutation S475A, Kan^r	This study
pET30a-PrcΔPEP	pET30a:: Prc, expressing full length Prc_{Xcc} with PEP domain deletion, Kan^r	This study
pET30a-PrcΔDUF	pET30a:: Prc, expressing full length Prc_{Xcc} with DUF domain deletion, Kan^r	This study
pET30a-PrcΔDUF^{S475A}	pET30a:: Prc^{S475A}, expressing full length Prc_{Xcc} with DUF domain deletion and active site mutation S475A, Kan^r	This study
pET30a-VgrS	pET30a::VgrS, expressing full length of VgrS, Kan^r	Lab collection
pET30a-VgrS^{H186A}	pET30a::VgrS, expressing full length of VgrS with active site substitution H186A, Kan^r	Lab collection
pET30a-VgrS$^{A9G-Q10A}$	pET30a::VgrS, expressing full length of VgrS with double substitutions of A9G and Q10A, Kan^r	This study
pET30a-3R-VgrS	pET30a:: 3R-VgrS, expressing full length of VgrS with three arginines were inserted into the N-terminal, Kan^r	This study
pET30a-4R-VgrS	pET30a:: 4R-VgrS, expressing full length of VgrS with four arginines were inserted into the N terminal, Kan^r	This study
pET30a-3×Flag-VgrS	pET30a:: 3×Flag-VgrS, expressing full length of VgrS with 3×Flag tag was inserted into the N-terminal, Kan^r	This study
pET30a-VgrS-sensor	pET30a::sensor, expressing sensor region of VgrS, Kan^r	Lab collection
pET30a-VgrS-sensor$^{A9G-Q10A}$	pET30a::sensor$^{A9G-Q10A}$, expressing sensor region of VgrS with double substitutions of A9G and Q10A, Kan^r	This study
pET30a-VgrS-3×Flag-sensor	pET30a:: 3×Flag-sensor, expressing sensor region of VgrS with 3×Flag tag was inserted into the N-terminal , Kan^r	This study
pMBP-$VgrS_{cyto}$	pMal-p2X::$VgrS_{cyto}$, expressing cytoplasmic portion of VgrS, Amp^r	Lab collection
pET30a-PhoQ	pET-30a::PhoQ, expressing sensor region of PhoQ, Kan^r	Lab collection

Supplementary Table 2. Primers used in this study

Primer nam	Sequence (forward/reverse)	Length of products/description
Δprc	A: TCTAGACTGGGCGAAACGATGGAG<br>B: GCATGCTGCATTGGTGTAGGCGTTGAG<br>C: GCATGCCAAGTACTGCCGCAATCGAC<br>D: AAGCTTGGCACTGATGAGCGTGGG	AB 1147 bp; CD 666 bp; for Δprc construction
V-Δprc	F: GCCCAAGACCCGGATCGAGA<br>R: TTGTTGGTGCTGCCTTCGCT	WT 3613 bp; Δprc 2146 bp; for verification of Δprc construction
ΔPDZ	A: TCTAGACAAACTGGTCTACGGGTTGCTCTCG<br>B: GCATGCCGACAGCGACATCTGCTGATTGAAG<br>C: GCATGCGTGCGCCTGGCCGAAC<br>D: AAGCTTACGCCCTTGTGCTGGGTG	AB 604 bp; CD 605 bp; for ΔPDZ construction
ΔPEP	A: TCTAGATGGGCAGCCGACGACAAG<br>B: GCATGCCCGGCGCTGTGGCACGTC<br>C: GCATGCGCCAGCGTGGATGCCAC<br>D: AAGCTTGCTCACCCATCAATGACGCA	AB 633 bp; CD 559 bp; for ΔPEP construction
ΔDUF	A: TCTAGACAGCGCCGGATCGGCATCAT<br>B: GCATGCGCTGGCCGGGAATGCGATGTC<br>C: GCATGCAAGGACCAGCCGCTGTCGGC<br>D: AAGCTTGGCACTGATGAGCGTGGG	AB 567 bp; CD 601 bp; for ΔDUF construction
V-ΔPDZ	F: GCATGCGGTGAGCACCAGTGCCAGCAGGC<br>R: GCATGCGCTGGCCGGGAATGCGATGTC	WT 1677 bp; ΔPDZ 1389 bp; for verification of ΔPDZ construction

V-ΔPEP	F: CAAACTGGTCTACGGGTTGCTCTCG<br>R: GGCACTGATGAGCGTGGG	WT 2588 bp; ΔPEP 2036 bp; for verification of ΔPEP construction
V-ΔDUF	F: GTGCGCCTGGCCGAAC<br>R: TTGTTGGTGCTGCCTTCGCT	WT 1871 bp; ΔDUF 1409 bp; for verification of ΔDUF construction
Δprc-prc	F: AAGCTTATGACCTACAACGTTTCTGCGT<br>R: GGTACCTCAGTCAGCCCAGCGACC	2184 bp; for *prc* complementation
Δprc-prc$_{Xoo}$	F: AAGCTTATGAAGGCCGGCCTGCTG<br>R: GGTACCTCAATCCGCCCAACGCCCCG	2160bp; for *prc* heterogenetic complementation
Δprc-prc-HA-Flag^{S475A}	F: AAGCTTATGACCTACAACGTTTCTGCGT<br>R: GGTACCTCACTTGTCGTCGTCGTCCTTGTAGTCGACCTTGAGAGCG TAATCTGGAACATCGTATGGGTAGTCAGCCCAGCGACCC	2244 bp; for overexpression prc-HA-Flag and prc-HA-Flag^{S475A}
prc^{S475A}	F: TGATCAACCGCGGCTCGGCTGCGGCGTCGGAAATT<br>R: CAGCCGAGCCGCGGTTGATCAGCACGCCCAGCG	for prc^{S475A} site mutation construction
prc^{K500A}	F: ATTGGTGAAACCACCTTCGGCGCGGGCACGGTGCAGAAC<br>R: GCGCCGAAGGTGGTTTCACCAATGACCAGACCGC	for prc^{K500A} site mutation construction
ΔvgrSΔ9	A: GGATCCCGACATCGCCGCCAATC<br>B: CATATGCATCACGTCCTCCACCAGCT<br>C: CATATGCAGCGGTTCTATTCCGATCCG<br>D: GCATGCGCCATCCTGGGTGTATTTGAC	AB 821 bp; CD 888 bp; for vgrS$^{\Delta 9}$ construction
V-ΔvgrSΔ9	F: CCGATGATCCAGACCCGTC	WT 253 bp; for

	R: TGCGAACGCGTCGATATTG	verification of vgrS$^{\Delta9}$ construction
ΔvgrS-vgrS^{A9G}	F: CCGCAATATCGACGCGTTCGGACAGCGGTTCTATTC<br>R: CCGAACGCGTCGATATTGCGGTTCAT	For ΔvgrS-vgrS with A9G site mutation construction
ΔvgrS-vgrS$^{A9G\text{-}Q10A}$	F: AATATCGACGCGTTCGGAGCGCGGTTCTATTCCG<br>R: GCTCCGAACGCGTCGATATTGCGGTTCAT	For ΔvgrS-vgrS with A9G-Q10A double mutation construction
3HA-vgrS^{A9G}	F: CGCTAATATCGACGCGTTCGGACAGCGGTTCTATTC<br>R: CCGAACGCGTCGATATTAGCGTAATCTGGAACATC	for 3HA-vgrS with A9G site mutation construction
3HA-vgrS$^{A9G\text{-}Q10A}$	F: CGCTAATATCGACGCGTTCGGAGCGCGGTTCTATTCCGA<br>R: GCTCCGAACGCGTCGATATTAGCGTAATCTGGAACATC	for 3HA-vgrS with A9G-Q10A double mutation construction
ΔvgrS-3HA-vgrS	F: GTCGACATGAACCGCTACCCATACGATGTTCCAGAT TACGCTAATATCGACGCGTTCGCAC<br>R: GAGCTCTTAGCGATGGAACGCCAGTG	1184 bp; for overexpression 3HA-vgrS
ΔvgrS-3×Flag-vgrS	F: GTCGACATGGACTACAAGGACGACGACGACAAGGACTACAAGGACGACGACGACAAGGACTACAAGGAC GACGACGACAAG AACCGCAATATCGACGCG<br>R: GAGCTCTTAGCGATGGAACGCCAGTG	1229 bp; for overexpression 3×Flag-vgrS
Δ0943	A: GAATTCCGTTCGCTACGCCTGTATG<br>B: GGATCCCTTGAGTTTGGCTTGATGGAA<br>C: GGATCCCTGACCGTGCTGCCCTAC	AB 319 bp; CD 373 bp; for Δ0943 construction

	D: AAGCTTCGCATTGGCTGGAGCAG	
Δ2164	A: GAATTCTTGCGTGGGAAGTATTGCG<br>B: GGATCCCTGCTCGGCCTGCTGCTC<br>C: GGATCCGCAAAGTGGCTGCTGGAACA<br>D: AAGCTTGACCACAGACCATGCCGATT	AB 320 bp; CD 344 bp; for Δ2164 construction
Δ0690	A: TCTAGAATGGGCAGAAAACGAAGTCAA<br>B: CATATGGTCGTAGGCGAGGGAACCAC<br>C: CATATGCGGCTGGAACGAGCAGGACA<br>D: AAGCTTGCCTCGCAGCAACCATTACA	AB 259 bp; CD 433 bp; for Δ0690 construction
Δ3300	A: GGATCCTCTTCACGCACCTGCCCAA<br>B: AAGCTTGCCGTCGATCGACCAGTTC<br>C: AAGCTTACCAAGAACGGCGTTGATGC<br>D: CTGCAGCGTGCTGCCAGTGCAGTTCG	AB bp; CD bp; for Δ3300 construction
Δ3301	A: GGATCCCATCCAGAGCCATCGGGG<br>B: AAGCTTGACCGGATTCACCGACACCG<br>C: AAGCTTATTCTCAATCGCGCGGCC<br>D: CTGCAGCACATCGCTACGGTGTCGATC	AB bp; CD bp; for Δ3301 construction
Δ3576	A: GGATCCGCGTGACGTCTATCACGCTT<br>B: AAGCTTCGCACTGGCGGAAAGG<br>C: AAGCTTTCGGCGATGTTGAACATGTC<br>D: CTGCAGTTGGCCGGCCAAGCCAC	AB 448 bp; CD 297 bp; for Δ3576 construction
V-Δ0943	F: AAGCGATTGATGCGGCG<br>R: GCTGTACCCGAACGTGGACT	WT 1363 bp; Δ0943 980 bp; for verification of Δ0943 construction
V-Δ2164	F: CCCGTAAGGGCGGAATAG<br>R: GCCACGATGTTACGGTTATTTT	WT 1240 bp; Δ2164 890 bp; for verification of Δ2164

		construction
V-Δ0690	F: CCACCGAGGGTTCGTTCA<br>R: CCGTGCTGAGTTGTCCCAC	WT 1597 bp; Δ0690 1034 bp; for verification of Δ0690 construction
V-Δ3300	F: CAATTCAGGTCGGGCGGGC<br>R: TAGGGCTGCGCCAGCACG	WT 1847 bp; Δ3300 1109 bp; for verification of Δ3300 construction
V-Δ3301	F: CGGCAGTGAGAATTTTCTTG<br>R: GAAGGGTGCTGTGCAT	WT 1876 bp; Δ3301 1153 bp; for verification of Δ3301 construction
V-Δ3576	F: GGTGGCTGTTTTCCAAAGA<br>R: TTGGGTCGGTTCCTTGA	WT 7631 bp; Δ3576 983 bp; for verification of Δ3576 construction
Pro-Prc	F: CATATGACCTACAACGTTTCTGCGTCCC<br>R: AAGCTTGTCAGCCCAGCGACCCG	2178 bp; for full length protein Prc expression
Pro-PDZ	F: CATATGCTGGAAGGCATCGGCG<br>R: AAGCTTCTTCTGCCGCGTCAGGG	288 bp; for expressing the PDZ domain of Prc
Pro-PEP	F: CATATGATCGGCATCATCAAACTGCC<br>R: AAGCTTCGGGAATGCGATGTCCG	552 bp; for expressing the

		PEP domain of Prc
Pro-DUF	F: CATATGGTGGATGCCACCGAATTC<br>R: AAGCTTGGACAACAACCCCAGCG	462 bp; for expressing the DUF domain of Prc
pro-PEP+DUF∆20	F: CATATGCAGGATTTCGAAGGCCGTCG<br>R: AAGCTTGGCCGCCGATTCGTGC	960 bp; for expressing the PEP+DUF$^{\Delta 20}$ domain of Prc
pro-∆PDZ	F: CATATGCAGGATTTCGAAGGCCGTCG<br>R: AAGCTTGGCCGCCGATTCGTGC	960 bp; for expressing the Prc without PDZ domain
pro-∆PEP	F: CATATGCAGGATTTCGAAGGCCGTCG<br>R: AAGCTTGGCCGCCGATTCGTGC	960 bp; for expressing the Prc without PEP domain
pro-∆DUF	F: CATATGCAGGATTTCGAAGGCCGTCG<br>R: AAGCTTGGCCGCCGATTCGTGC	960 bp; for expressing the Prc without DUF domain
pro-3R-VgrS	F: GCTCATATGCGACGACGAATGAACCGCAATATCGACGC<br>R: CCGCTCGAGGCGATGGAACGCCAGTGTTG	1167 bp; for the 3R-VgrS expression
pro-4R-VgrS	F: GCTCATATGCGACGACGACGAATGAACCGCAATATCGACGC<br>R: GCTCATATGCGACGACGACGAATGAACCGCAATATCGACGC	1170 bp; for the 4R-VgrS expression
pro-3×Flag-VgrS	F:	1230 bp; for the

	CATATGGACTACAAGGACGACGACGACAAGGACTACAAGGACGACGACGACAAGGACTACAAGGACGACGACGACAAG ATGAACCGCAATATCGACGC<br>R: GCTCATATGCGACGACGACGAATGAACCGCAATATCGACGC	3×Flag -VgrS expression
pro-VgrS-sensor	F: CATATGAACCGCAATATCGACGC<br>R: CTCGAGGGTGCGCTTGAGCTGGA	297 bp; for the VgrS sensor expression
pro-VgrS-sensor^{A9G}	F: CGCAATATCGACGCGTTCGGACAGCGGTTCTATTC<br>R: CCGAACGCGTCGATATTGCGGTTCATATG	for VgrS sensor with A9G mutation expression
pro-VgrS-sensor$^{A9G-Q10A}$	F: GCAATATCGACGCGTTCGGAGCGCGGTTCTATTCCGAT<br>R: GCTCCGAACGCGTCGATATTGCGGTTCATATG	for VgrS sensor with A9G-Q10A double site mutation expression
pro-3×Flag-VgrS-sensor	F:<br>CATATGGACTACAAGGACGACGACGACAAGGACTACAAGGACGACGACGACAAGGACTACAAGGACGACGACGACAAG ATGAACCGCAATATCGACGC<br>R: CCGCTCGAGGGTGCGCTTGAGCTGGATC	273 bp; for the 3×Flag –VgrS sensor expression
qRT-Prc	F: TGTGGCGGCAGTCGGTGAT<br>R: GCATTGGTGTAGGCGTTGAGGA	169 bp; for for real time PCR analysis to check the expression level of *prc*
qRT-0943	F: CAACCGCGAGATCGAAGACG<br>R: GCTGGAATGGGCTGAGGAA	128 bp; for for real time PCR analysis to check the expression level of *XC_0943*
qRT-2164	F: AAGATGGCTGCCTTTGGAC<br>R: CACATTCGCTGGGTTTCG	164 bp; for real time PCR analysis to check the expression level of

		yciE
qRT-3300	F: AAGTGCCCGACCTCCCA<br>R: TGCCCTTCGAGTCGGTGT	208 bp; for real time PCR analysis to check the expression level of *XC_3300*
qRT-3301	F: CGATGCCACTGACCACGCTTAC<br>R: TCTGCCGGACCCACTCAACG	210 bp; for real time PCR analysis to check the expression level of *XC_3301*
tmRNA	F: CCAACGACGACAACTACGGT<br>R: ACGAGCACGGGCACAAG	111 bp for real time PCR as internal control
P0711	F: CAGTTGGGTGGTCTGTCCG<br>R: CCGCCCATTATCGGGC	362 bp; probe for EMSA, also for ChIP-qPCR amplification
P0943	F: CGTTCGCTACGCCTGTAT<br>R: CCCAGTCTGAACCTGACT	186 bp; probe for EMSA, also for ChIP-qPCR amplification
P2164	F: AGGCAGCGACAAGCCAGTG<br>R: CGGGGTCTCCTGTTGTCTACG	205 bp; probe for EMSA, also for ChIP-qPCR amplification
P3300	F: ACTTGGACACGGTCTTCACG<br>R: GTATACAGCTCCTTCTTTGGGAGAT	309 bp; probe for EMSA
P3301	F: GCGTTAACGGTCATATAACATGTGA<br>R: CGGAGCGCTCCTAGTGGG	301 bp; probe for EMSA
P0769	F: ACCCCACCGGTATCTTCA	236 bp; unspecific

	R: CAGCATGTAGCCCTCGTT	probe for EMSA
0710-a<br>0711-b	CATGTGGGAAGTCGAAGACG<br>AGCAGTTCAACGCCGGTAG	335bp; for 0710-0711 intergenic transcript identification
0711-c<br>0712-d	ACTCCTCCAGTGGCAAATACGT<br>TGCATTGCACGCCAGACC	213bp; for 0711-0712 intergenic transcript identification
0712-e<br>0713-f	AACCGTTCCACCGCATCAA<br>TGTCGCCACCGATCTGCTT	299bp; for 0712-0713 intergenic transcript identification
0713-g<br>Prc-h	CCGATGGTGCGTTCCTCGTA<br>TTGGTCGCCGTCGCCTGTT	420bp; for 0713-Prc intergenic transcript identification
Prc-i<br>0715-j	GAATCGGCGGCCATCCT<br>GCGTGTTCTTCGGCACCTT	280bp; for Prc-0715 intergenic transcript identification
Prc-k<br>Prc-l	GTGGATGCCACCGAATTC<br>GGACAACAACCCCAGCG	462bp; for operon identification, positive control

Supplementary Table 3. Proteins identified by tandem-affinity purification (TAP) under osmostress-treated or untreated conditions

Protein code[a]	Annotation or description	Score[b]	Theoretical MW(Da)[C]	Osmostress
**1. Transport Protein**				
XC_1141	Translocation protein TolB	20.18	47034.7	Treated
**2. Signal Transduction**				
vgrS	Two-component system sensor protein	80.26	42693.2	Untreated
XC_3452	Two-component system regulatory protein	20.19	28107.1	Untreated
**3. Transcription and Translation**				
XC_3317	30S ribosomal protein S4	30.16	23280.7	Treated
XC_0829	L-isoaspartate protein carboxylmethyltransferase	30.24	24078.5	Untreated
XC_2863	30S ribosomal protein S2	30.29	30168.3	Untreated
XC_3342	Elongation factor Tu	50.23	43171.2	Untreated/Treated
XC_3354	Elongation factor Tu	50.23	43185.2	Untreated/Treated
**4. Cell Structures and Division**				
XC_1619	Oar protein	20.24	117750.6	Untreated
XC_1625	PilY1 protein	20.24	130606.9	Untreated
XC_3122	Septum site-determining protein	30.17	28938.0	Untreated
XC_3300	Outer membrane protein	30.24	39362.0	Untreated/Treated
XC_2958	Beta-hexosaminidase	130.33	34515.9	Untreated/Treated
**5. Fatty Acid Metabolism**				
XC_3652	Beta-ketoacyl-[ACP] synthase I	40.27	41937.2	Untreated
XC_0851	Inorganic pyrophosphatase	20.16	19712.4	Untreated

**6. Amino Acid Metabolism**				
XC_3358	Ribose-phosphate pyrophosphokinase	20.22	34513.7	Untreated
**7. Nucleotide Metabolism**				
XC_3346	DNA-directed RNA polymerase subunit beta'	30.15	155280.8	Treated
XC_3347	DNA-directed RNA polymerase subunit beta	20.15	154862.6	Treated
XC_2346	Carbamoyl phosphate synthase large subunit	30.18	117276.3	Untreated
XC_2534	CTP synthetase	20.18	61513.7	Untreated
XC_2348	Carbamoyl phosphate synthase small subunit	30.22	40264.5	Untreated
XC_2509	Recombinase A	20.18	37017.4	Untreated
XC_3316	DNA-directed RNA polymerase subunit alpha	20.16	36363.3	Untreated
**8. Carbohydrate Metabolism**				
XC_0972	Glyceraldehyde-3-phosphate dehydrogenase	20.13	35984.9	Treated
XC_1578	Acetyl-CoA carboxylase subunit beta	24.15	32013.7	Treated
XC_2977	3-hydroxyisobutyrate dehydrogenase	20.22	29510.9	Untreated
XC_1930	UTP-glucose-1-phosphate uridylyltransferase	20.22	32101.6	Untreated
XC_0979	Fructose-bisphosphatealdolase	20.20	36276.2	Untreated
XC_3627	GDP-mannose 4,6-dehydratase	90.28	38744.9	Untreated/Treated
**9. Energy Metabolism**				
XC_1595	NADH dehydrogenase subunit G	40.27	79082.8	Untreated/Treated
XC_3678	ATP synthase F0F1 subunit beta	30.22	50996.5	Untreated/Treated
XC_3680	ATP synthase F0F1 subunit alpha	20.23	55323.6	Untreated/Treated
**10. Small Molecules Metabolism**				

XC_0713	Lipoyl synthase	50.26	36895.9	Untreated
XC_1845	N-ethylammelinechlorohydrolase	20.24	48513.0	Untreated
XC_2761	pyridoxine kinase	40.26	31915.2	Untreated/Treated
**11. Degradative Enzymes**				
XC_0256	Acyl-CoA dehydrogenase	20.13	41503.3	Untreated
XC_0714	Tail-specific protease	340.31	79988.6	Untreated/Treated
**12. Protein Maintenance and Folding**				
XC_2763	Molecular chaperone DnaK	20.16	68836.8	Treated
XC_0535	Molecular chaperone GroEL	20.18	57183.9	Untreated/Treated
**13. Oxidation-Reduction Process**				
XC_3396	Alkyl hydroperoxide reductase	20.15	20432.1	Untreated
**14. Virulence, Resistance, and Adaptation**				
XC_0009	Biopolymer transport ExbB protein	20.19	26666.2	Untreated
**15. Hypothetical Protein**				
XC_0538	Hypothetical protein XC_0538	50.24	100325.1	Treated
XC_4310	Hypothetical protein XC_4310	30.20	89711.1	Untreated
XC_0215	Hypothetical protein XC_0215	30.20	21846.7	Untreated/Treated

[a] Protein code is based on the genomic annotation of *X. campestris* pv. *campestris* 8004.
[b] Score is the result of SEQUEST search, Score > 20 was regarded as significant difference ($P < 0.05$).
[c] MW: molecular weight.

Supplementary Table 4. ChIP-seq analysis identifies genes with promoter regions bound by VgrR in *X. campestris* pv. *campestris* stimulated by osmostress

Gene[a]	Gene Function	Strand	Score	Peak Start:Peak End	Peak position	Direction
	**WT-vgrR-his$_6$**					
**01. Transport Protein**						
**XC_0849**	TonB-dependent receptor	+	241	1025491:1025790	1025641	ui
**02. Signal Transduction**						
**XC_0850**	response regulator	-	188	1030551:1030850	1030701	u
**XC_1049**	two-component system regulatory protein	+	396	1265632:1265931	1265782	i
**03. Transcription, Translation, and Modification**						
**XC_4364**	Thr tRNA	-	194	3695521:3695820	3695671	u
**XC_3230**	50S ribosomal protein L32	-	238	3875863:3876162	3876013	ui
**XC_3308**	peptidyl-prolyl cis-trans isomerase	-	186	3962296:3962595	3962446	ui
**XC_3309**	GTP-binding elongation factor protein	+	186	3962296:3962595	3962446	u
**XC_3438**	peptidyl-prolyl cis-trans isomerase	+	231	4088083:4088382	4088233	ui
**XC_3843**	RNA polymerase sigma-32 factor	-	206	4541163:4541462	4541313	i
**XC_4383**	23sRNA	-	247	4632753:4633052	4632903	i
**XC_3945**	arginine--tRNA ligase	+	211	4658019:4658318	4658169	i
**XC_4390**	23sRNA	-	242	5020267:5020566	5020417	i
**XC_4393**	16sRNA	-	251	5023342:5023641	5023492	d
**04. Cell Structures and Division**						
**XC_0691**	rod shape-determining protein	+	179	829695:829994	829845	u
**XC_0937**	fimbrial assembly protein	-	191	1126440:1126739	1126590	i

**XC_0941**	fimbrial assembly membrane protein	-	324	1129598:1129897	1129748	ui
**XC_0942**	penicillin-binding protein 1A	+	324	1129598:1129897	1129748	u
**XC_0969**	outer membrane protein	-	216	1163156:1163455	1163306	u
**XC_1619**	Oar protein	+	288	1940371:1940670	1940521	ui
**XC_2869**	undecaprenyl pyrophosphate synthetase	+	181	3449055:3449354	3449205	ui
**XC_3259**	murein hydrolase D	+	215	3900468:3900767	3900618	i
**XC_3278**	UDP-2,3-diacylglucosamine hydrolase	+	191	3925724:3926023	3925874	i
**XC_3300**	outer membrane protein	-	232	3952153:3952452	3952303	ui
**XC_3504**	UDP-3-O-[3-hydroxymyristoyl] N-acetylglucosamine deacetylase	-	383	4162419:4162718	4162569	d
**XC_3515**	penicillin-binding protein 3	-	184	4178501:4178800	4178651	u
**XC_3576**	outer membrane protein	-	289	4250782:4251081	4250932	u
**XC_3814**	lipopolysaccharide core biosynthesis glycosyl transferase	+	201	4508266:4508565	4508416	ui
**05. Carbohydrate Metabolism**						
**XC_0493**	bacterioferritin-associated ferredoxin	-	225	581363:581662	581513	d
**XC_0690**	sugar kinase	-	179	829695:829994	829845	ui
**XC_0972**	glyceraldehyde-3-phosphate dehydrogenase	+	189	1164704:1165003	1164854	ui
**XC_0979**	fructose-bisphosphate aldolase	+	195	1174888:1175187	1175038	ui
**XC_3251**	3-deoxy-D-manno-octulosonic acid kinase	-	202	3894583:3894882	3894733	u
**06. Energy metabolism**						
**XC_3082**	cytochrome O ubiquinol oxidase subunit III	-	208	3691863:3692162	3692013	u
**XC_3604**	flavoprotein-ubiquinone oxidoreductase	+	197	4283446:4283745	4283596	u
**XC_3680**	ATP synthase alpha chain	-	223	4368610:4368909	4368760	i
**07. Amino Acid Metabolism**						

**XC_0530**	3-dehydroquinate dehydratase	-	217	626188:626487	626338	u
**XC_1608**	30S ribosomal protein S15	+	204	1930173:1930472	1930323	ui
**XC_3544**	serine hydroxymethyltransferase	-	197	4207201:4207500	4207351	i
**XC_3635**	cystathionine gamma-lyase-like protein	-	198	4318076:4318375	4318226	i
**08. Nucleotide Metabolism**						
**XC_0265**	nuclease	+	215	318762:319061	318912	u
**XC_0589**	RNA-directed DNA polymerase	+	213	704416:704715	704566	u
**XC_1609**	polynucleotide phosphorylase	+	196	1932036:1932335	1932186	i
**XC_1614**	phosphoribosylaminoimidazole carboxylase ATPase subunit	+	198	1936388:1936687	1936538	ui
**XC_2749**	oxoglutarate dehydrogenase	+	188	3302860:3303159	3303010	i
**XC_3244**	DNA polymerase III tau and gamma subunits	-	187	3889889:3890188	3890039	u
**XC_3282**	amidophosphoribosyltransferase	-	211	3930951:3931250	3931101	ui
**XC_RS21975**	RNase_P_RNA	-	201	4182477:4182776	4182627	ui
**XC_3603**	DNA repair system specific for alkylated DNA	-	197	4283446:4283745	4283596	ui
**09. Small Molecules Metabolism**						
**XC_0713**	lipoic acid synthetase	+	208	856218:856517	856368	i
**XC_2653**	imidazolonepropionase	-	200	3192301:3192600	3192451	ui
**XC_2654**	atrazine chlorohydrolase	+	200	3192301:3192600	3192451	ui
**XC_4102**	reductase/halogenase	+	212	4829154:4829453	4829304	u
**11. Degradive Enzymes**						
**XC_0714**	tail-specific protease	+	312	856865:857164	857015	u
**XC_3195**	integral membrane proteinase subunit	-	184	3830111:3830410	3830261	u
**XC_3377**	extracellular protease	-	216	4028304:4028603	4028454	u

**XC_3832**	protease IV	-	202	4528630:4528929	4528780	i
**XC_4009**	peptidase	-	202	4727419:4727718	4727569	i
**12. Regulatory Function**						
**XC_0486**	CRP-like protein Clp	-	205	575741:576040	575891	i
**XC_3720**	leucine responsive regulatory protein	+	192	4407079:4407378	4407229	u
**XC_3966**	transcriptional regulator tetR family	+	237	4683390:4683689	4683540	ui
**13. Pathogenicity, Virulence, and Adaptation**						
**XC_0639**	cellulase	-	211	766711:767010	766861	i
**XC_1141**	TolB protein	+	209	1383632:1383931	1383782	i
**XC_3608**	phosphoglucomutase; phosphomannomutase	+	203	4289410:4289709	4289560	i
**XC_3809**	phosphinothricin acetyltransferase	-	203	4503968:4504267	4504118	u
**XC_4223**	OmpA-related protein	+	231	4992575:4992874	4992725	u
**14. Mobile Genetic Elements**						
**XC_2777**	phage-related protein	-	400	3334306:3334605	3334456	d
**15. Oxidation-Reduction Process**						
**XC_2164**	ferritin-like domain-containing protein	-	281	2610300:2610599	2610450	u
**XC_3301**	oxidoreductase	+	232	3952153:3952452	3952303	ui
**16. Hypothetical Protein**						
**XC_0943**	hypothetical protein	+	265	1132766:1133065	1132916	ui
**XC_1011**	conserved hypothetical protein	-	311	1223678:1223977	1223828	ui
**XC_1012**	conserved hypothetical protein	+	311	1223678:1223977	1223828	u
**XC_1068**	conserved hypothetical protein	-	212	1286065:1286364	1286215	ui
**XC_1242**	conserved hypothetical protein	-	263	1522269:1522568	1522419	ui

**XC_1306**	conserved hypothetical protein	-	197	1597230:1597529	1597380	ui
**XC_1534**	conserved hypothetical protein	-	378	1843408:1843707	1843558	ui
**XC_1620**	conserved hypothetical protein	+	181	1944784:1945083	1944934	i
**XC_2931**	hypothetical protein	+	283	3516867:3517166	3517017	ui
**XC_3113**	conserved hypothetical protein	-	192	3725301:3725600	3725451	u
**XC_3238**	conserved hypothetical protein	+	201	3882586:3882885	3882736	u
**XC_3434**	conserved hypothetical protein	+	201	4082880:4083179	4083030	u
**XC_3556**	conserved hypothetical protein	-	273	4222497:4222796	4222647	ui
**XC_3595**	conserved hypothetical protein	+	194	4274020:4274319	4274170	u
**XC_3690**	conserved hypothetical protein	-	208	4377907:4378206	4378057	u
**XC_3813**	conserved hypothetical protein	-	201	4508266:4508565	4508416	u
**XC_3823**	hypothetical protein	+	225	4518103:4518402	4518253	u
**XC_3956**	conserved hypothetical protein	+	228	4674222:4674521	4674372	u
**XC_4181**	conserved hypothetical protein	+	208	4937298:4937597	4937448	u

Δprc-vgrR-his$_6$

**Gene**	**Protein name**	**Strand**	**Score**	**Peak Start:Peak End**	**Summits**	**Location**
**01. Transport Protein**						
**XC_0849**	TonB-dependent receptor	+	227	1025553:1025852	1025703	ui
**XC_3295**	ABC transporter sulfate binding protein	-	165	3947788:3948087	3947938	ui
**02. Signal Transduction**						
**XC_0850**	response regulator	-	176	1029349:1029648	1029499	i
**XC_1049**	two-component system regulatory protein	+	394	1265615:1265914	1265765	i
**XC_1062**	two-component system sensor protein	-	180	1280539:1280838	1280689	u

**03. Transcription, Translation, and Modification**						
**XC_3230**	50S ribosomal protein L32	-	231	3875838:3876137	3875988	ui
**XC_4366**	Ser tRNA	-	183	3890144:3890443	3890294	i
**XC_3254**	protein phosphatase	+	179	3896492:3896791	3896642	ui
**XC_4367**	Ser tRNA	-	179	3896492:3896791	3896642	u
**XC_3261**	peptidyl-prolyl cis-trans isomerase	-	200	3904937:3905236	3905087	u
**XC_3266**	peptidyl-prolyl cis-trans isomerase	-	208	3911808:3912107	3911958	u
**XC_3308**	peptidyl-prolyl cis-trans isomerase	-	198	3962364:3962663	3962514	ui
**XC_3309**	GTP-binding elongation factor protein	+	198	3962364:3962663	3962514	ui
**XC_3363**	peptide chain release factor 1	+	169	4012671:4012970	4012821	u
**XC_3643**	ATP-dependent RNA helicase	+	182	4325445:4325744	4325595	u
**XC_3806**	RNA polymerase sigma-70 factor	-	178	4501300:4501599	4501450	ui
**XC_3819**	polypeptide deformylase	-	195	4515189:4515488	4515339	ui
**XC_3843**	RNA polymerase sigma-32 factor	-	196	4541144:4541443	4541294	i
**XC_3889**	30S ribosomal protein S21	+	210	4593876:4594175	4594026	ui
**XC_4393**	16sRNA	-	251	5023403:5023702	5023553	d
**04. Cell Structures and Division**						
**XC_0691**	rod shape-determining protein	+	182	829641:829940	829791	u
**XC_0695**	rod shape-determining protein	+	192	835672:835971	835822	i
**XC_0941**	fimbrial assembly membrane protein	-	322	1129599:1129898	1129749	ui
**XC_0942**	penicillin-binding protein 1A	+	322	1129599:1129898	1129749	ui
**XC_0970**	outer membrane protein	-	190	1163330:1163629	1163480	i
**XC_3300**	outer membrane protein	-	236	3952162:3952461	3952312	ui

**XC_3517**	cell division protein	-	168	4179047:4179346	4179197	i
**XC_3576**	outer membrane protein	-	307	4250778:4251077	4250928	u
**XC_3617**	aminotransferase	+	164	4297853:4298152	4298003	i
**XC_3718**	alanine racemase	-	171	4405847:4406146	4405997	ui
**05. Carbohydrate Metabolism**						
**XC_0588**	pyruvate dehydrogenase	+	177	703656:703955	703806	i
**XC_0690**	sugar kinase	-	182	829641:829940	829791	u
**XC_0978**	pyruvate kinase type II	+	167	1172841:1173140	1172991	ui
**XC_1063**	succinyl-CoA synthetase beta subunit	+	180	1280539:1280838	1280689	ui
**XC_3268**	isocitrate dehydrogenase	-	181	3914586:3914885	3914736	u
**XC_3607**	3-oxoadipate CoA-succinyl transferase alpha subunit	-	187	4289011:4289310	4289161	u
**XC_3613**	dTDP-glucose-4,6-dehydratase	-	184	4295440:4295739	4295590	u
**XC_3689**	dihydrolipoamide dehydrogenase	-	178	4377252:4377551	4377402	u
**06. Energy metabolism**						
**XC_1597**	NADH-ubiquinone oxidoreductase NQO9 subunit	+	173	1916840:1917139	1916990	i
**XC_1602**	NADH-ubiquinone oxidoreductase NQO14 subunit	+	174	1922548:1922847	1922698	i
**XC_3083**	cytochrome O ubiquinol oxidase subunit I	-	184	3693390:3693689	3693540	i
**XC_3084**	cytochrome O ubiquinol oxidase subunit II	-	170	3694941:3695240	3695091	u
**XC_3614**	electron transfer flavoprotein beta subunit	+	184	4295440:4295739	4295590	ui
**XC_3680**	ATP synthase alpha chain	-	191	4368872:4369171	4369022	i
**07. Amino Acid Metabolism**						
**XC_0530**	catabolic dehydroquinase	-	176	625922:626221	626072	ui
**XC_3544**	serine hydroxymethyltransferase	-	186	4207544:4207843	4207694	ui

**08. Nucleotide Metabolism**						
**XC_0265**	nuclease	+	196	318525:318824	318675	u
**XC_1609**	polynucleotide phosphorylase	+	161	1931995:1932294	1932145	i
**XC_2749**	oxoglutarate dehydrogenase	+	167	3301159:3301458	3301309	i
**XC_2766**	recombination protein N	+	159	3322931:3323230	3323081	u
**XC_3282**	amidophosphoribosyltransferase	-	185	3930802:3931101	3930952	ui
**XC_3442**	diadenosine tetraphosphatase	+	190	4092368:4092667	4092518	i
**XC_RS21975**	RNase_P_RNA	-	177	4182381:4182680	4182531	ui
**XC_3578**	phosphoribosylformylglycinamidine synthetase	-	182	4254785:4255084	4254935	i
**XC_3596**	prolyl-tRNA synthetase	+	162	4274914:4275213	4275064	ui
**XC_3945**	arginyl-tRNA synthetase	+	176	4657601:4657900	4657751	ui
**09. Small Molecules Metabolism**						
**XC_0529**	biotin carboxyl carrier protein of acetyl-CoA	-	176	625922:626221	626072	u
**XC_0712**	lipoate biosynthesis protein B	+	179	854823:855122	854973	ui
**XC_2653**	imidazolonepropionase	+	187	3192289:3192588	3192439	ui
**XC_2654**	atrazine chlorohydrolase	-	187	3192289:3192588	3192439	ui
**XC_3229**	beta-ketoacyl-[ACP] synthase III	-	231	3875838:3876137	3875988	u
**XC_3433**	2-octaprenyl-6-methoxyphenol hydroxylase	-	169	4083075:4083374	4083225	ui
**XC_3439**	pyridoxal phosphate biosynthetic protein	+	167	4089669:4089968	4089819	i
**XC_3642**	fumarylacetoacetate hydrolase	-	182	4325445:4325744	4325595	u
**10. Protein Maintenance and Folding**						
**XC_0535**	60 kDa chaperonin	+	164	633603:633902	633753	i
**XC_2931**	heat-shock protein Hsp70	+	294	3516859:3517158	3517009	u

**11. Degradative Enzymes**						
**XC_2772**	serine peptidase	-	191	3330847:3331146	3330997	u
**XC_3196**	integral membrane protease subunit	-	175	3830927:3831226	3831077	ui
**XC_3379**	extracellular protease	-	184	4029346:4029645	4029496	i
**XC_3585**	aminopeptidase A/I	+	189	4261880:4262179	4262030	ui
**XC_3832**	protease IV	-	170	4528695:4528994	4528845	i
**XC_3888**	O-sialoglycoprotein endopeptidase	-	210	4593876:4594175	4594026	u
**12. Regulatory Function**						
**XC_0830**	transcriptional regulator	-	180	997816:998115	997966	ui
**XC_1309**	nitrogen regulatory IIA protein	-	185	1600188:1600487	1600338	ui
**XC_2253**	transcriptional regulator	+	223	2711165:2711464	2711315	u
**XC_3966**	transcriptional regulator tetR family	+	197	4683393:4683692	4683543	ui
**13. Pathogenicity, Virulence, and Adaptation**						
**XC_2227**	HrpX related protein	+	343	2687926:2688225	2688076	i
**XC_3608**	phosphoglucomutase; phosphomannomutase	+	187	4289011:4289310	4289161	ui
**XC_4223**	OmpA-related protein	+	197	4992565:4992864	4992715	u
**XC_4296**	toluene tolerance protein	+	244	5097673:5097972	5097823	ui
**14. Mobile Genetic Elements**						
**XC_2777**	phage-related protein	-	400	3334294:3334593	3334444	d
**15. Oxidation-Reduction Process**						
**XC_3301**	oxidoreductase	+	236	3952162:3952461	3952312	u
**16. Hypothetical Protein**						
**XC_0831**	conserved hypothetical protein	+	180	997816:998115	997966	ui

**XC_1011**	conserved hypothetical protein	-	335	1223681:1223980	1223831	ui
**XC_1012**	conserved hypothetical protein	+	335	1223681:1223980	1223831	u
**XC_1068**	conserved hypothetical protein	-	173	1286056:1286355	1286206	ui
**XC_1143**	conserved hypothetical protein	+	199	1384855:1385154	1385005	u
**XC_1242**	conserved hypothetical protein	-	244	1522241:1522540	1522391	u
**XC_1306**	conserved hypothetical protein	-	184	1597264:1597563	1597414	u
**XC_1534**	conserved hypothetical protein	-	378	1843392:1843691	1843542	ui
**XC_2810**	conserved hypothetical protein	-	188	3383673:3383972	3383823	ui
**XC_2811**	conserved hypothetical protein	+	188	3383673:3383972	3383823	u
**XC_3238**	conserved hypothetical protein	+	187	3882600:3882899	3882750	u
**XC_3257**	conserved hypothetical protein	-	195	3899681:3899980	3899831	u
**XC_3434**	conserved hypothetical protein	+	169	4083075:4083374	4083225	u
**XC_3503**	conserved hypothetical protein	+	389	4162407:4162706	4162557	d
**XC_3540**	conserved hypothetical protein	+	207	4203436:4203735	4203586	ui
**XC_3556**	conserved hypothetical protein	-	277	4222468:4222767	4222618	ui
**XC_3584**	conserved hypothetical protein	-	189	4261880:4262179	4262030	u
**XC_3637**	UptF protein	-	195	4320457:4320756	4320607	u
**XC_3820**	conserved hypothetical protein	+	195	4515189:4515488	4515339	u
**XC_3822**	Smg protein	+	199	4517649:4517948	4517799	ui
**XC_3956**	conserved hypothetical protein	+	220	4674355:4674654	4674505	ui
**XC_4295**	conserved hypothetical protein	-	244	5097673:5097972	5097823	u
**XC_4311**	conserved hypothetical protein	-	187	5117949:5118248	5118099	u

a. Promoters identified by the ChIP-seq when bacterium was treated with osmostress. Genes in blue indicate that its 5′ upstream regions were identified in both strains WT-vgrR-his_6 and Δprc-vgrR-his_6.

Supplementary References

1. Wang, L. et al. Two-component signaling system VgrRS directly senses extracytoplasmic and intracellular iron to control bacterial adaptation under iron depleted stress. *PLoS Pathog.* **12** (2016).
