# Supplementary material for: Proteolysis of histidine kinase VgrS inhibits its autophosphorylation and promotes osmostress resistance in Xanthomonas campestris
Source: Nat Commun. 2018 Nov 15;9:4791. doi: 10.1038/s41467-018-07228-4 (PMC6237974; doi:10.1038/s41467-018-07228-4)
Supplement: Supplementary file 3 — Reporting Summary [file 41467_2018_7228_MOESM3_ESM.pdf]

nature research

Corresponding author(s): Wei Qian

Reporting Summary

Nature Research wishes to improve the reproducibility of the work that we publish. This form provides structure for consistency and transparency in reporting. For further information on Nature Research policies, see Authors & Referees and the Editorial Policy Checklist.

Statistical parameters

When statistical analyses are reported, confirm that the following items are present in the relevant location (e.g. figure legend, table legend, main text, or Methods section).

n/a	Confirmed	
☐	☒	The exact sample size (*n*) for each experimental group/condition, given as a discrete number and unit of measurement
☐	☒	An indication of whether measurements were taken from distinct samples or whether the same sample was measured repeatedly
☐	☒	The statistical test(s) used AND whether they are one- or two-sided<br>*Only common tests should be described solely by name; describe more complex techniques in the Methods section.*
☒	☐	A description of all covariates tested
☒	☐	A description of any assumptions or corrections, such as tests of normality and adjustment for multiple comparisons
☐	☒	A full description of the statistics including central tendency (e.g. means) or other basic estimates (e.g. regression coefficient) AND variation (e.g. standard deviation) or associated estimates of uncertainty (e.g. confidence intervals)
☐	☒	For null hypothesis testing, the test statistic (e.g. *F*, *t*, *r*) with confidence intervals, effect sizes, degrees of freedom and *P* value noted<br>*Give P values as exact values whenever suitable.*
☒	☐	For Bayesian analysis, information on the choice of priors and Markov chain Monte Carlo settings
☒	☐	For hierarchical and complex designs, identification of the appropriate level for tests and full reporting of outcomes
☒	☐	Estimates of effect sizes (e.g. Cohen's *d*, Pearson's *r*), indicating how they were calculated
☐	☒	Clearly defined error bars<br>*State explicitly what error bars represent (e.g. SD, SE, CI)*

Our web collection on statistics for biologists may be useful.

Software and code

Policy information about availability of computer code

Data collection	No specific code or software was used in data collection
Data analysis	Microsoft Excel 2010 was used to perform the Student's t-test in this work

For manuscripts utilizing custom algorithms or software that are central to the research but not yet described in published literature, software must be made available to editors/reviewers upon request. We strongly encourage code deposition in a community repository (e.g. GitHub). See the Nature Research guidelines for submitting code & software for further information.

Data

Policy information about availability of data

All manuscripts must include a data availability statement. This statement should provide the following information, where applicable:
- Accession codes, unique identifiers, or web links for publicly available datasets
- A list of figures that have associated raw data
- A description of any restrictions on data availability

All relevant data in this work has been included in the manuscript. The original, uncropped images were shown in Supplementary Fig. 10. Data and experimental materials are available from the corresponding author upon reasonable request. The accession number for the ChIP-seq dataset reported in this study is GenBank GEO: GSE120292 [https://www.ncbi.nlm.nih.gov/geo/query/acc.cgi?acc=GSE120292].

April 2018

Field-specific reporting

Please select the best fit for your research. If you are not sure, read the appropriate sections before making your selection.

- [x] Life sciences
- [ ] Behavioural & social sciences
- [ ] Ecological, evolutionary & environmental sciences

For a reference copy of the document with all sections, see nature.com/authors/policies/ReportingSummary-flat.pdf

Life sciences study design

All studies must disclose on these points even when the disclosure is negative.

Sample size	The sample size of all the experiments are beyond three samples or more. the number of n is indicated in the figure legends.
Data exclusions	No data was excluded in the analysis.
Replication	All experiments were repeated three or two times.
Randomization	The experiment design and data collection are randomized.
Blinding	Investigators are blinding to the experimental results

Reporting for specific materials, systems and methods

Materials & experimental systems

n/a	Involved in the study	
☒	☐	Unique biological materials
☐	☒	Antibodies
☒	☐	Eukaryotic cell lines
☒	☐	Palaeontology
☒	☐	Animals and other organisms
☒	☐	Human research participants

Methods

n/a	Involved in the study	
☐	☒	ChIP-seq
☒	☐	Flow cytometry
☒	☐	MRI-based neuroimaging

Antibodies

Antibodies used	We used polyclonal antiserum of VgrS, Prc, HPPK and monoclonal antibodies for HA (M20003-L, Abmart, China) and His6 tags (M20001L, Abmart, China)
Validation	Yes, these antibodies were verified by Western blotting using purified proteins.

ChIP-seq

Data deposition

- [x] Confirm that both raw and final processed data have been deposited in a public database such as GEO.
- [x] Confirm that you have deposited or provided access to graph files (e.g. BED files) for the called peaks.

Data access links<br>*May remain private before publication.*	https://www.ncbi.nlm.nih.gov/geo/query/acc.cgi?acc=GSE120292
Files in database submission	GSE120292_RAW.tar, GSM3397750_WT_CvgrR-his_treat_afterfiting_NC_007086.1.wig.gz, GSM3397751_DLTPrc_CvgrR-his_treat_afterfiting_NC_007086.1.wig.gz
Genome browser session<br>(e.g. UCSC)	No longer applicable

Methodology

Replicates	The experimental replicates are 3, the sample of the three replicates was mixed for high-throughput sequencing

Sequencing depth	Sequencing depth is 1G, the total number of reads is 6275823, length of reads is 101bp and they were paired-end
Antibodies	anti-His6 antibody (Abmart, #M20001, lot: 294073)
Peak calling parameters	Peak calling was conducted by MACS2
Data quality	The high-throughput sequencing reads were analysed using the Burrows–Wheeler Aligner method
Software	The consensus binding motif analysis was completed with MEME and FIMI tools in the MEME software suite
